# Sliding behaviour and surface quality after static air polishing of conventional and modern bracket materials: In vitro analysis

**DOI:** 10.1007/s00056-021-00352-9

**Published:** 2021-09-23

**Authors:** Lutz Hodecker, Christoph Bourauel, Bert Braumann, Teresa Kruse, Hildegard Christ, Sven Scharf

**Affiliations:** 1 Department of Orthodontics, University Hospital of Heidelberg, Im Neuenheimer Feld 400, 69120 Heidelberg, Germany; 2 Department of Oral Technology, University of Bonn, Welschnonnenstraße 17, 53111 Bonn, Germany; 3 Department of Orthodontics, University of Cologne, Faculty of Medicine and University Hospital Cologne, Kerpener Straße 32, 50931 Cologne, Germany; 4 Institute of Medical Statistics and Computational Biology (ISMB), University of Cologne, Robert-Koch-Straße 10, 50931 Cologne, Germany

**Keywords:** Sliding resistance, 3D-printed brackets, Powder polishing, Slot precision, Professional tooth cleaning, Gleitwiderstand, 3D-gefertigte Brackets, Pulverpolitur, Präzision der Slots, Professionelle Zahnreinigung

## Abstract

**Objectives:**

As part of orthodontic treatment, air polishing is routinely used for professional tooth cleaning. Thus, we investigated the effects of static powder polishing on sliding behaviour and surface quality of three different bracket materials (polymer, ceramic, metal), including a 3D-printed bracket.

**Methods:**

Two bracket types of each material group were polished with an air-polishing device using sodium bicarbonate. Exposure times were set at 10, 20, and 60 s; the application distance was 5 mm. The force loss due to sliding resistance was tested with an orthodontic measurement and simulation system (OMSS) using a 0.016 inch × 0.022 inch stainless steel archwire. Untreated brackets served as control. Polishing effects and slot precision were evaluated using an optical digital and scanning electron microscope.

**Results:**

Sliding behaviour and slot precision differed significantly between and within the groups. Prior to polishing, polymer brackets showed the least force loss, ceramic brackets the highest. With progressive polishing time, the resistance increased significantly with titanium brackets (26 to 37%) and decreased significantly with steel brackets (36 to 25%). Polymer brackets showed the smallest changes in force loss with respect to polishing duration. Slot precision showed the largest differences between material groups and was primarily manufacturer-dependent with hardly any changes due to the polishing time.

**Conclusion:**

Powder polishing can positively or negatively affect the sliding properties of the bracket–archwire complex but is more dependent on the bracket–archwire material combination (i.e., manufacture-dependent slot precision). For titanium brackets, resistance only increased after 60 s of polishing. For ceramic brackets, effective reduction was observed after 10 s of polishing. Polymer brackets, including the 3D-printed brackets, showed better sliding properties than ceramic or metal brackets even after polishing for 60 s. Removal of plaque and dental calculus should lead to a noticeable improvement of the sliding properties and outweighs structural defects that may develop.

## Abstract (de)

**Zielsetzungen:**

Im Rahmen einer kieferorthopädischen Behandlung wird das Air-Polishing routinemäßig zur professionellen Zahnreinigung eingesetzt. Wir untersuchten daher die Auswirkungen des statischen Pulverpolierens auf das Gleitverhalten und die Oberflächenqualität von 3 unterschiedlichen Bracketmaterialien (Polymer, Keramik, Metall), einschließlich eines 3‑D-gedruckten Brackets.

**Methoden:**

Zwei Brackettypen aus jeder Materialgruppe wurden mit einem Druckluftpoliergerät unter Verwendung von Natriumbicarbonat poliert. Die Expositionszeiten wurden auf 10, 20 und 60 s festgelegt; der Applikationsabstand betrug 5 mm. Der Kraftverlust durch den Gleitwiderstand wurde mit einem kieferorthopädischen Mess- und Simulationssystem (OMSS) unter Verwendung eines 0,016 inch × 0,022 inch Edelstahlbogens getestet. Unbehandelte Brackets dienten als Kontrolle. Die Auswirkungen des Polierens und die Präzision der Slots wurden mit einem optischen Digital- und Rasterelektronenmikroskop untersucht.

**Ergebnisse:**

Das Gleitverhalten und die Präzision der Slots unterschieden sich signifikant zwischen den Gruppen und innerhalb der Gruppen. Vor dem Polieren zeigten Polymerbrackets den geringsten Kraftverlust, Keramikbrackets den höchsten. Mit zunehmender Polierdauer nahm der Widerstand bei Titanbrackets signifikant zu (26–37%), bei Stahlbrackets signifikant ab (36–25%). Polymerbrackets zeigten die geringsten Veränderungen des Kraftverlusts in Abhängigkeit von der Polierdauer. Die Slot-Präzision wies die größten Unterschiede zwischen den Materialgruppen auf und war in erster Linie herstellerabhängig, wobei es kaum Veränderungen durch die Polierdauer gab.

**Schlussfolgerung:**

Das Pulverpolieren kann sich positiv oder negativ auf die Gleiteigenschaften des Bracket-Bogen-Draht-Komplexes auswirken, ist jedoch stärker von der Bracket-Bogen-Draht-Materialkombination abhängig (d. h. von der herstellerabhängigen Slot-Präzision). Bei Titanbrackets erhöhte sich der Widerstand erst nach 60 s Polieren. Bei Keramikbrackets wurde eine wirksame Reduzierung nach 10 s Polieren beobachtet. Polymerbrackets, einschließlich der 3D-gedruckten Brackets, wiesen selbst nach 60 s Polieren noch bessere Gleiteigenschaften auf als Keramik- oder Metallbrackets. Die Entfernung von Plaque und Zahnstein sollte zu einer erkennbaren Verbesserung der Gleiteigenschaften führen und wiegt die möglicherweise entstehenden strukturellen Defekte auf.

## Introduction

Orthodontic patients are subject to difficult oral hygiene when wearing fixed appliances. Thus, regular motivations interviews and professional tooth cleaning by means of air polishing are important measures to prevent white spot lesions [4, 20, 30]. Various methods for professional tooth cleaning have been established, of which mainly air-powder or rubber cup polishing systems are used. Comparative studies have shown that tooth cleaning with air-powder polishing seems to be more efficient than with a polishing rubber cup [8]. When using air-polishing devices, glycine or sodium bicarbonate appliances are used to remove plaque, staining or dental calculus, whereby a different field of application is recommended for both types of powder. The glycine powder consists of smaller particle size (25 µm) and is considered less abrasive. It is therefore more suitable for the subgingival use in the context of periodontal therapy, for example, cleaning exposed roots, dentin surfaces or gingival pockets of smaller size [6, 25]. The use of sodium bicarbonate powder (40 µm) has been established for supragingival enamel surface cleaning. Due to the relatively long duration of orthodontic treatment, professional tooth cleaning should be carried out regularly to prevent the development of white spot lesions. For this reason, knowledge of possible disadvantages of this cleaning process on orthodontic treatment is important. Earlier studies have also dealt with this topic [19, 37]. It was the intention of the study presented here to analyse polished brackets in combination with unpolished archwires. This was done because of the assumption that in the majority of prophylaxis appointments an archwire change will be performed. Professional tooth cleaning is carried out more easily and efficiently in most cases if the archwire is first removed from the brackets. In addition, it was found that the presence of plaque and dental calculus in the bracket–archwire complex affects the efficiency of an orthodontic treatment by increasing the sliding resistance [10]. Thus, clean surfaces of the bracket–archwire complex are desirable. Smooth surfaces of brackets and archwires allow low friction sliding behaviour and result in better implementation of the planned tooth movement. In addition, low resistance in sliding mechanics correlate with shortened treatment time and a reduction of several side effects, such as root resorption or the appearance of white spot lesions [20, 35]. For this reason, procedures that could lead to a significant increase of surface roughness and thus of resistance should be avoided.

The aim of this study was to determine the mechanical effects of powder polishing of varying duration on the surface roughness of the bracket slot and thus on the sliding behaviour of several bracket–archwire combinations, including a modern 3D-printed polymer bracket filled with aluminiumoxide ceramic. It was of interest to determine whether a possible limitation in the number or manner of the air-polishing process should be recommended.

## Materials and methods

**Fig. 1:**
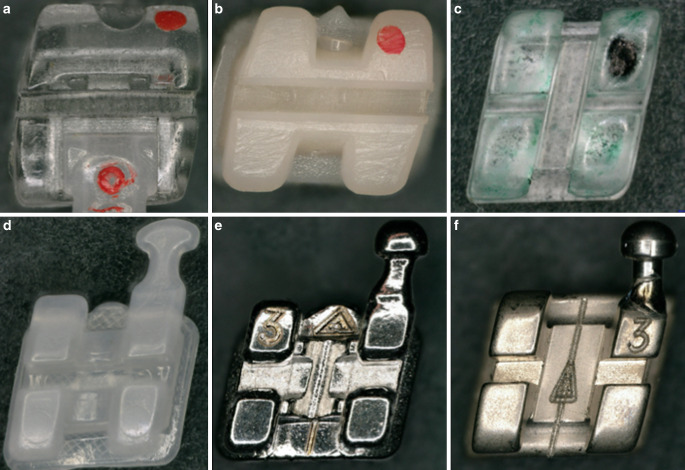
Overview of the examined bracket types with the polymer group: 3D-printed Shark SL (**a**) and Brillant® bracket (**b**), the ceramic group: Inspire Ice™ (**c**) and discovery® pearl bracket (**d**) and the metal group: discovery® (**e**) and equilibrium® ti bracket (**f**) Übersicht über die untersuchten Brackettypen mit der Polymergruppe: 3D-gedrucktes Shark SL (**a**) und Brillant®-Bracket (**b**), der Keramikgruppe: Inspire Ice™ (**c**) und discovery® pearl Bracket (**d**) und der Metallgruppe: discovery® (**e**) und equilibrium® ti Bracket (**f**)

Two bracket types from three material groups (polymer, ceramic and metal) were selected for this study (Fig. 1). All brackets were for tooth 23 with similar slot size type (0.018 inch) and torque value (0°) and included the following: for the polymer group—the Brillant® bracket (injection moulded polyoxymethylene, Forestadent Bernhard Förster GmbH, Pforzheim, Germany) as well as the 3D-printed self-ligating Shark SL bracket (Dentalline GmbH & Co. KG, Birkenfeld, Germany); for the ceramic group—the discovery® pearl (Dentaurum GmbH & Co. KG, Ispringen, Germany) and the Inspire Ice™ Bracket (Ormco Europe, BR Amersfoort, The Netherlands); and for the metal group—the discovery® and equilibrium® ti bracket (Dentaurum GmbH & Co. KG, Ispringen, Germany).

For the first experimental part, the air-polishing device AirFlow® Master (EMS Electro Medical Systems GmbH, Munich, Germany) filled with AirFlow® classic powder (sodium bicarbonate, 40 µm, EMS Electro Medical Systems GmbH, Munich, Germany) was used. The test brackets were glued on metal plates of 5 × 5 mm size (DeguDent GmbH, Hanau-Wolfgang, Germany) and positioned exactly 5 mm below the nozzle of the Air-Flow® device in accordance with the guidelines of EMS. This was realised by using a fixed and windowed acrylic template. The window within this template had exactly the dimensions for the fitting of the metal plates. The handpiece was fixed over a laboratory stand and directed at a 45° angle to the mesial side of the bracket slot. Thus, standardised conditions could be established for all test runs. The locking mechanism of the self-ligating 3D-printed Shark SL bracket was opened and fixed permanently with adhesive before polishing. This was done in order to establish equal conditions between all bracket types because all other brackets were of the conventionally ligating types. Before each test run, the pressure in the powder chamber of the air-polishing device was checked using a barometer and regulated to 2.8 bar. Both the powder and the quantity of the liquid were set to an average value. The tested brackets were statically polished for 10, 20, and 60 s and then cleaned with cold water and dried with an air blower. Assuming that a bracket is polished for a maximum of 5 s during a professional tooth cleaning, a polishing time of 60 s corresponds to 12 treatments of a quarterly basis over a period of 3 years. The polishing time was controlled with a stopwatch. All polishing experiments were repeated with four other similar brackets, so that a total of five brackets per type were polished under the same conditions. This sample size was chosen from the experience of previous studies using the orthodontic measurement and simulation system (OMSS). The unpolished brackets served as controls (0 s).

**Fig. 2:**
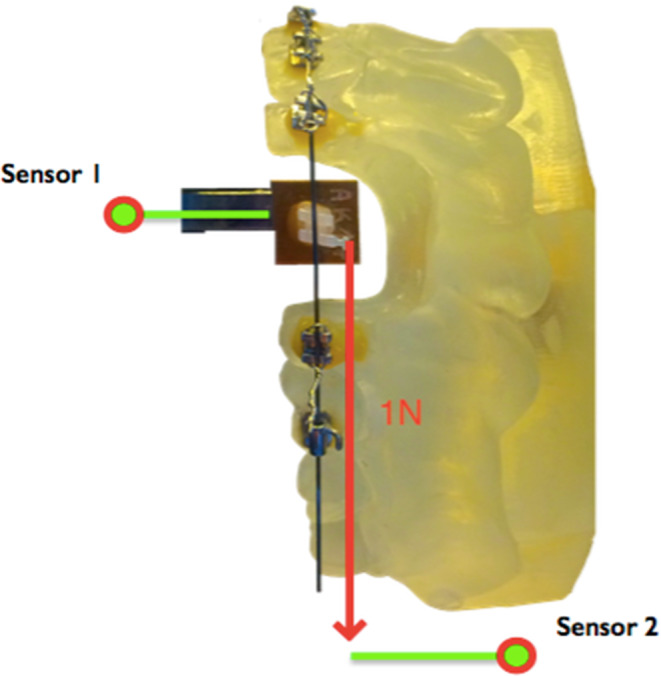
Schematic illustration of the orthodontic measurement and simulation system (OMSS) Schematische Darstellung des kieferorthopädischen Mess- und Simulationssystems (OMSS)

The second experimental part dealt with the resistance measurements. The change of the surface quality of all bracket slots after polishing was determined by measuring the force loss due to resistance with the help of the OMSS. This apparatus simulates orthodontic tooth movement by applying a specific orthodontic force [14]. It records the occurring forces at the test brackets three-dimensionally via force/torque sensors. A resin replica of an upper jaw model by Frasaco (Frasaco GmbH, Tettnang, Germany) was used for fixing the archwires, in which tooth 23 was replaced by a test bracket. Tooth 24 also had to be removed to ensure a distalization path. The model as well as the brackets were mounted in the OMSS in such a way that initially no forces were measurable. Only then was the experimental force applied to the test bracket. The bracket was linked via an arm structure to the first sensor of the OMSS for measuring the occurring forces. A second sensor was used to measure the level of the applied force, implemented by connecting a nickel–titanium spring coil (rematitan®LITE; Dentaurum GmbH & Co. KG, Ispringen, Germany) to both the first sensor (via the hook of the experimental bracket 23) and the second sensor (Fig. 2). When simulating an orthodontic tooth movement, the resulting force loss due to resistance was calculated by subtracting the force level at the bracket sensor from the orthodontic force that was applied. In this example, a distalizing force of 1 N was applied with the help of the nickel–titanium spring coil. Each test bracket was uniformly ligated according to the recommendations of Schumacher et al. [31]. They determined that the ligature process has a significant influence on the sliding behaviour between bracket and archwire. Therefore, the ligature (remanium® preformed ligature 0.010 inch; Dentaurum GmbH & Co. KG, Ispringen, Germany) was closed and then reopened with a 180° turn until no forces and torques were measurable in the simulation system. Following these preconditions, the measurements started with distalizing the test bracket, which means that the test bracket combined with the first sensor was moved towards the second sensor by spring force. The positioning accuracy of the stepper motors was in the range of 1 µm/0.01°, whereas the measuring accuracy of the sensors was in the range of 0.02 N for force measurement and 0.5 Nmm for torque measurement. In the context of this distalization, 200 measured values of force loss were noticed and recorded in a software developed for these types of experiments as well as in the program Microsoft Excel (Microsoft Corporation, Redmond, WA, USA) for further analyses. All brackets were measured with a spring hardened stainless steel archwire of 0.016 inch × 0.022 inch cross-section size (remanium®, Dentaurum GmbH & Co. KG, Ispringen, Germany).

**Fig. 3:**
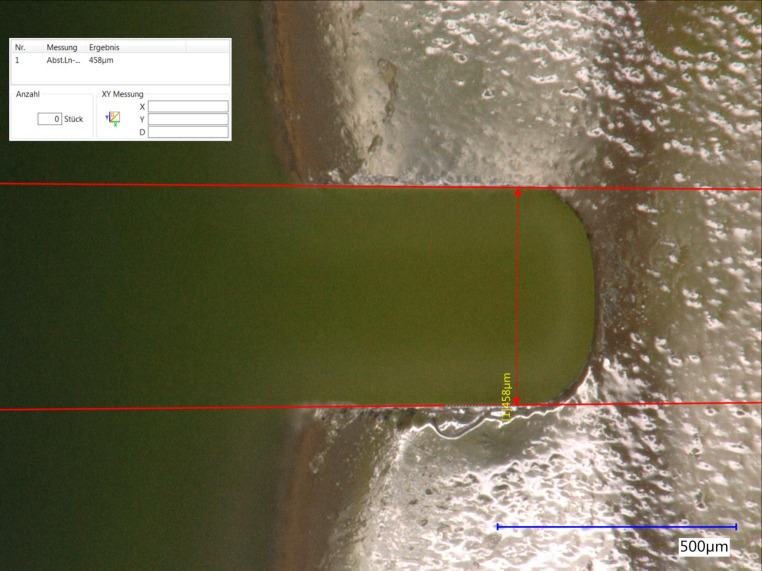
Measurement of the vertical dimension of the mesial bracket slot at the example of Inspire Ice™ ceramic brackets. Light microscope image, 200× magnification Messung der vertikalen Dimension des mesialen Bracketslots am Beispiel der Inspire Ice™ Keramikbrackets. Lichtmikroskopische Aufnahme, Vergr. 200:1

In the third experimental part of this investigation, the bracket slots were measured and optically inspected for defects using the Keyence® VHX500 optical digital microscope as well as a scanning electron microscope (Amray 1610T, Bedford, MA, USA). For the digital microscopic measurements of the mesial slot dimensions, all brackets were tared in such a way that the vertical limitations of the mesial slot could be unequivocally determined (Fig. 3). This was done in order to interpret the measured force loss values because of the fact that resistance behaviour depends decisively on the slot precision. The microscope analysing software was used to draw lines for these limitations in order to measure the distance between them as an extent of slot precision. For each bracket type, the mean was calculated from the measurements of five different test brackets for each polishing time.

### Statistical analysis

Based on the 200 individual measured force loss values of each test bracket, the mean value and standard deviation were calculated for all tested bracket–archwire combinations to obtain a single force loss value for every test bracket. Each group consisted of 5 sample brackets for which the median, mean, and standard deviation were calculated. Here, the median values were used for the statistical tests. Because of the fact that a normal distribution of the results cannot be assumed for a sample size of 5, nonparametric statistical tests were used. Thus, the Kruskal–Wallis H test followed by the Mann–Whitney U test were applied to point out statistically relevant significances between the different groups. A significance level of 0.05 was defined for all evaluations as statistically significant. The statistical evaluation was undertaken with the Statistical Package for Social Sciences, version 25.0 (IBM, Armonk, NY, USA).

## Results

### Slot dimension

**Fig. 4:**
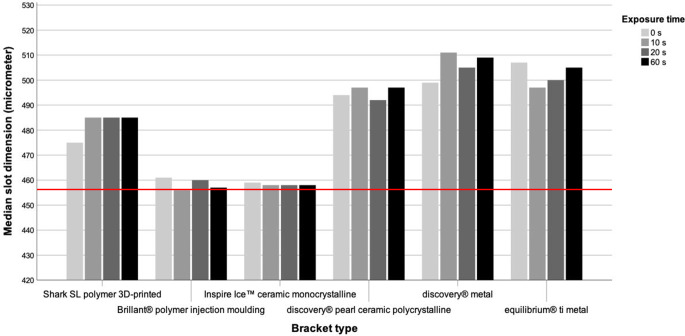
Diagram of the vertical slot dimension of all bracket types with respect to exposure time. The *red line* represents the DIN standard of 457 µm (0.018 inch) as the reference Diagramm der vertikalen Slotgröße aller Brackettypen in Bezug auf die Expositionszeit. Die *rote Linie* stellt die DIN-Norm von 457 µm (0,018 inch) als Referenz dar

The digital measurements of the slot walls in incisal–apical (vertical) direction pointed out that although all bracket types were within the DIN standards, they still showed clearly visible differences among each other (Fig. 4; Table 1). The smallest deviations from the DIN standards for orthodontic brackets and tubes (DIN 13971-2) of 457 µm were found for the Inspire Ice™ bracket as well as the Brilliant® bracket and turned out to be 1–2%. With a deviation of about 4% from the required dimension, the 3D-printed Shark SL bracket also showed rather high manufacturing precision. A deviation of about 7 to 10% was measured for the discovery® pearl, discovery® and equilibrium® ti brackets. A clearly identifiable effect of powder polishing on the vertical dimensions of the mesial bracket slot could not be detected.

**Table 1:** Measurements of the vertical slot dimension of all bracket types with respect to exposure time Messungen der vertikalen Slotdimension aller Brackettypen in Abhängigkeit von der Expositionszeit

	Slot dimension (µm)																			
	0 s					10 s					20 s					60 s				
	25%	Median	75%	Mean	SD	25%	Median	75%	Mean	SD	25%	Median	75%	Mean	SD	25%	Median	25%	Mean	SD
*Bracket type*																				
Shark SL	470	475	479	474	5	481	485	496	488	10	477	485	496	486	11	474	482	491	482	9
Brillant®	461	461	462	461	1	446	456	461	454	8	457	460	466	461	6	456	457	462	458	3
Inspire Ice™	458	459	461	459	2	457	458	460	458	2	457	458	462	459	4	456	458	459	457	2
Discovery® pearl	491	494	496	493	3	493	497	500	496	4	489	492	500	494	6	497	497	498	497	1
Discovery®	499	499	502	500	2	509	511	516	512	5	493	505	507	501	8	504	509	510	507	4
Equilibrium® ti	504	507	510	507	3	495	497	545	515	40	493	500	505	499	6	502	505	508	505	4

*SD* standard deviation

### Resistance behaviour and surface quality

**Fig. 5:**
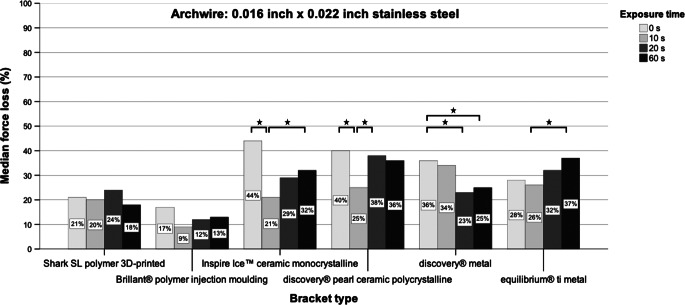
Force losses due to sliding resistance of all bracket types in combination with the 0.016 inch × 0.022 inch stainless steel archwire with respect to exposure time. The *stars* represent statistical significance (*p* ≤ 0.05, Mann–Whitney U test) Kraftverluste durch Gleitwiderstand aller Brackettypen in Kombination mit dem 0,016 inch × 0,022 inch Edelstahlbogen in Abhängigkeit von der Expositionszeit. Die *Sternchen* stehen für statistische Signifikanz (*p* ≤ 0,05, Mann-Whitney-U-Test)

In some cases, significant differences in measured force loss values could be observed. The exposure time as well as the combination of bracket and archwire material proved to be influencing variables (Fig. 5; Table 2).

**Table 2:** Measurements of the force losses due to sliding resistance of bracket types in combination with the 0.016 inch × 0.022 inch stainless steel archwire and with respect to exposure time Messungen der Kraftverluste aufgrund des Gleitwiderstands von Brackettypen in Kombination mit dem 0,016 inch × 0,022 inch Edelstahldraht und in Abhängigkeit von der Expositionszeit

	Force loss due to sliding resistance (%)																			
	0 s					10 s					20 s					60 s				
	25%	Median	75%	Mean	SD	25%	Median	75%	Mean	SD	25%	Median	75%	Mean	SD	25%	Median	75%	Mean	SD
*Bracket type*																				
Shark SL	13	21	23	18	6	10	20	30	20	11	24	24	33	27	5	15	18	25	19	6
Brillant®	8	17	20	14	7	4	9	12	8	4	6	12	21	13	9	9	13	15	12	3
Inspire Ice™	34	44	56	45	13	12	21	30	21	9	27	29	42	33	9	27	32	38	32	7
Discovery® pearl	37	40	49	42	7	19	25	26	23	4	29	38	51	39	12	28	36	42	35	8
Discovery®	34	36	39	36	3	28	34	42	35	10	19	23	31	24	8	22	25	30	26	4
Equilibrium® ti	22	28	54	36	17	21	26	30	25	5	24	32	41	32	9	31	37	43	37	7

*SD* standard deviation, Mann–Whitney U test

### Polymer brackets

**Fig. 6:**
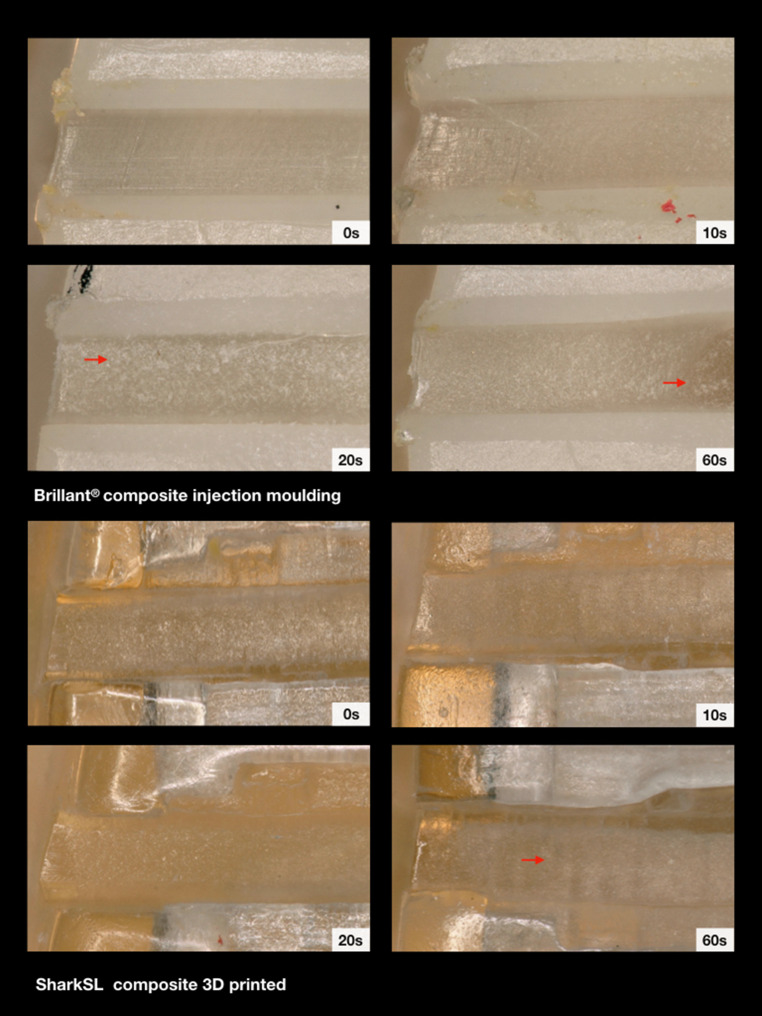
Illustration of the mesial slot properties of both polymer brackets with respect to exposure time. Increasing exposure time resulted in slight abrasion (*arrows*) which was mainly observed in the Brillant® brackets. Light microscope images 200× magnification Darstellung der mesialen Slot-Eigenschaften der beiden Polymerbrackets in Abhängigkeit von der Expositionszeit. Mit zunehmender Expositionszeit kam es zu leichtem Abrieb (*Pfeile*), der vor allem bei den Brillant®-Brackets zu beobachten war. Lichtmikroskopische Aufnahme, Vergr. 200:1

The comparison of the force loss values of the polished and unpolished 3D-printed polymer Shark SL brackets with those of the ceramic and metal group revealed the least values for the Shark SL brackets. No obvious effect of powder polishing on force loss was observed. The light and scanning electron microscope images of the brackets without polishing showed a rather smooth and plane slot surface (Figs. 6 and 9). After polishing, a general, slight roughening could be detected, whereas extensive defects or substance erosions were not observed. Only a few little slide lines from the archwire were found in the mesial slot area (Fig. 6).

When regarding the polished and the unpolished Brillant® polymer brackets, a rather consistent low resistance behaviour was also found when compared with the ceramic and metal brackets. As a minimally identifiable trend, the resistance decreased after 10 s of polishing time and increased again after 60 s. The light and scanning electron microscope images showed an overall roughening of the slot surface with increasing exposure time (Figs. 6 and 9). In addition, the light microscope images revealed clearly visible bracket damage caused by powder polishing. Curvy deformations of the slot wall or distinct perforations of the slot bottom were observed in several test brackets, mainly in the brackets after 60 s of exposure time. However, unpolished and polished brackets showed a kind of material abrasion or material shift at the mesial slot area, probably caused by the sliding movement of the archwire during distalization.

### Ceramic brackets

**Fig. 7:**
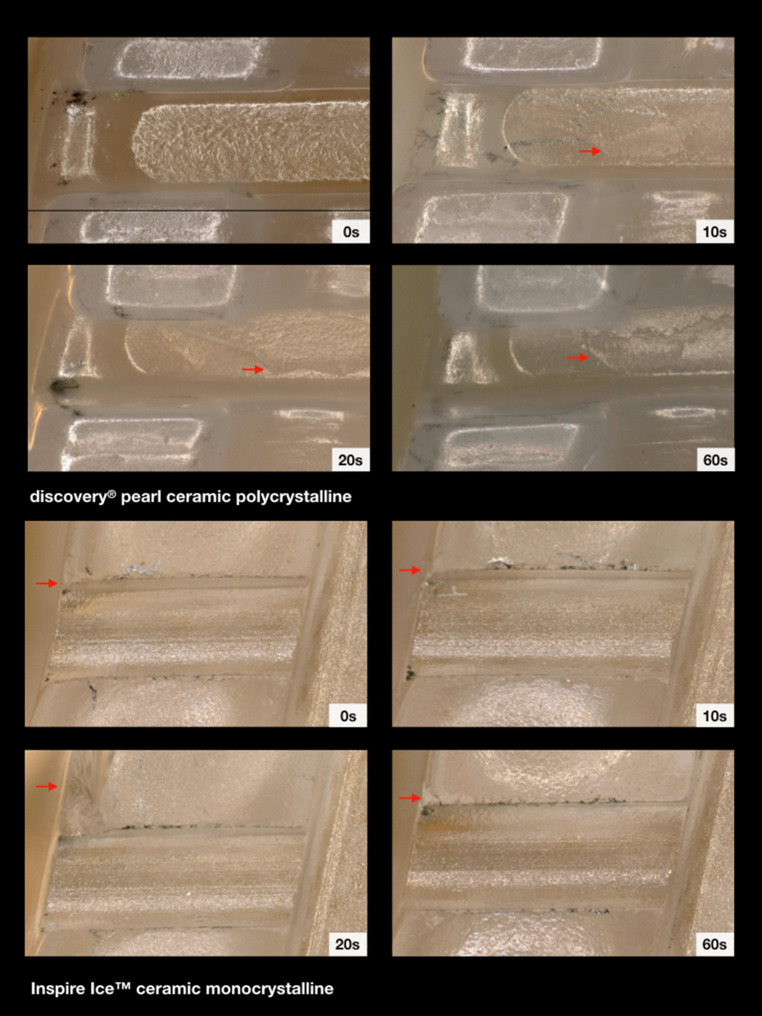
Illustration of the mesial slot properties of both ceramic brackets with respect to exposure time. Chipping effects (*arrows*) were clearly visible. Light microscope images, 200× magnification Darstellung der mesialen Slot-Eigenschaften der beiden Keramikbrackets in Abhängigkeit von der Expositionszeit. Chipping-Effekte (*Pfeile*) waren deutlich sichtbar. Lichtmikroskopische Aufnahme, Vergr. 200:1

The polished and unpolished Inspire Ice™ ceramic brackets revealed the highest force loss values compared to all other bracket types. Polishing for 10 s led to a significant reduction of the force loss level. However, the force loss levels subsequently increased with progressing exposure time. The light microscopic images showed large chipping effects at the mesial slot area in polished and even in unpolished brackets (Fig. 7). The electron scanning images showed a clear roughening of the slot surface with circular changes after 60 s of exposure time, possibly caused by the powder grains (Fig. 9).

The polished and unpolished discovery® pearl ceramic brackets also showed higher force loss levels compared to the polymer and metal group. The examination of the polished brackets pointed out a resistance reducing effect after polishing which means that the highest resistance levels were found initially and without polishing. The least force loss levels were observed after 10 s, which turned out to be statistically significant. After 20–60 s of polishing, the force loss values increased again but did not reach the initial amount of the unpolished brackets. Regarding the light microscope images, less chipping as seen as for the Inspire Ice™ ceramic brackets was detected for this ceramic bracket type. Only overall roughening or slight abrasion areas on the slot bottom were observed, mainly after 60 s of polishing (Fig. 7). The electron scanning images showed a rather smooth surface before polishing, whereas after an exposure of 60 s, a longish striped roughening was observed, which could also be caused by the impact of the powder grains (Fig. 9).

### Metal brackets

**Fig. 8:**
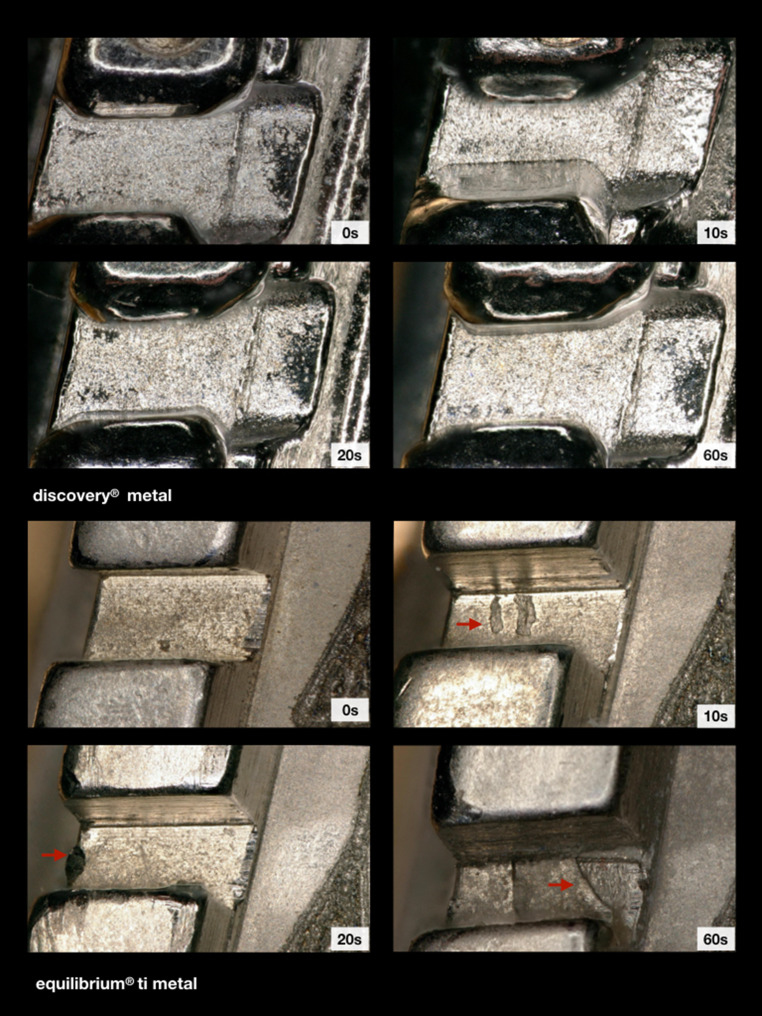
Illustration of the mesial slot properties of both metal brackets with respect to exposure time. Chipping effects (*arrows*) were mainly observed for the titanium bracket. Light microscope images, 200× magnification Darstellung der mesialen Slot-Eigenschaften der beiden Metallbrackets in Abhängigkeit von der Expositionszeit. Chipping-Effekte (*Pfeile*) wurden hauptsächlich beim Titan-Bracket beobachtet. Lichtmikroskopische Aufnahme, Vergr. 200:1

**Fig. 9:**
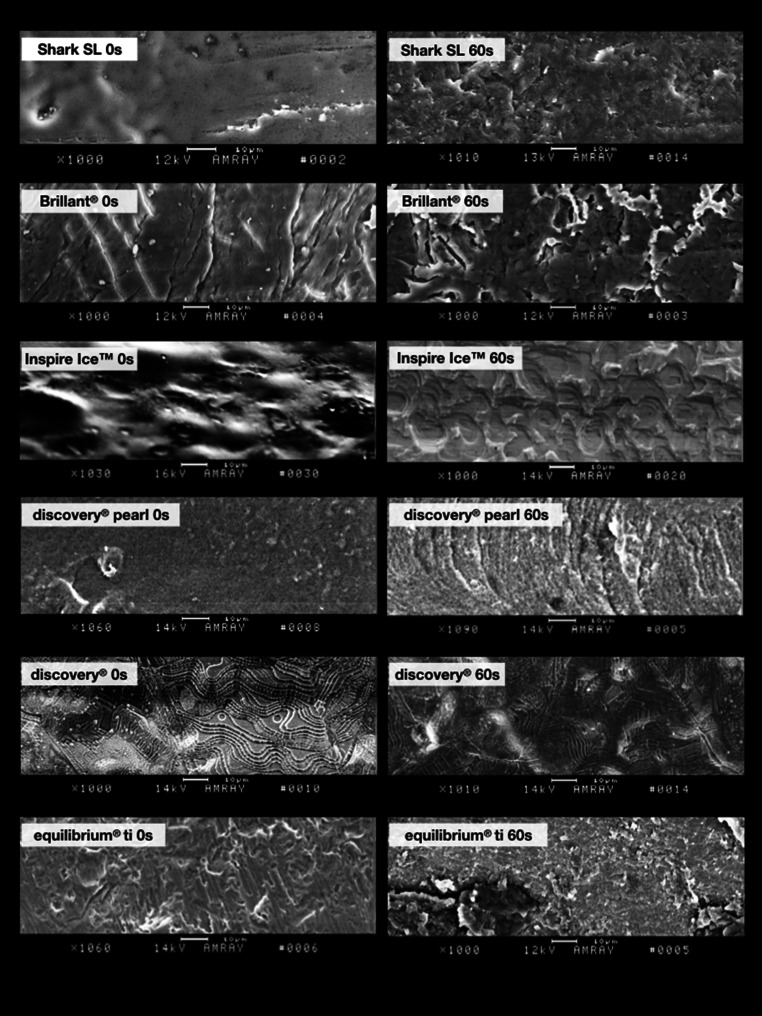
Scanning electron microscope images of all bracket types, before and after 60 s of exposure. 1000× magnification Rasterelektronenmikroskopische Aufnahmen aller Brackettypen, vor und nach 60 s Exposition. Vergr. 1000:1

The polished and unpolished discovery® metal brackets revealed less force loss values than the ceramic group, but higher levels than the polymer group. They did not show any characteristic resistance changes after 10, 20 or 60 s of polishing like other bracket types did. Thus, a clearly resistance-reducing or resistance-increasing trend could not be identified when increasing the exposure time. For this bracket type, no bracket damage after powder polishing was detected in the light microscopy images (Fig. 8). The electron scanning microscope images of the unpolished brackets revealed a surface pattern consisting of linear and circular patterns in a disordered arrangement. After an exposure time of 60 s this pattern was still recognizable, but in a much weaker and lighter version (Fig. 9).

The polished and unpolished titanium equilibrium® ti brackets showed slightly less force loss values compared to the other brackets of the ceramic and metal group, but also demonstrated higher levels than those from the polymer group. With regard to the unpolished brackets, a trend towards an increasing resistance after 60 s of polishing was observed, statistically significant for a polishing time of 20 s versus that of 60 s. The light microscope images illustrated sporadic damage in the sense of abrasion which could be found at the slot entrance as well as on the slot bottom (Fig. 8). The electron scanning microscope images showed a rougher surface and isolated abrasions after polishing (Fig. 9).

## Discussion

The present study provides a comparative overview of the mechanical and structural properties of different bracket materials before and after powder polishing with an Air-Flow® device. There are several studies that deal with the different resistance behaviour of unpolished metal and ceramic [2, 16], ceramic [21, 36] and polymer [15, 17] brackets or the difference between conventional and self-ligating brackets [26, 32–34]. Only a few have compared all three bracket material groups under the same conditions such as mechanical stress in the sense of powder polishing. Furthermore, a modern 3D-printed ceramic reinforced polymer bracket was also included in this study and compared to the other more common brackets.

At first, it was of interest to compare the sliding properties of the brackets without mechanical manipulation in the sense of powder polishing. Thus, the resistance values of the unpolished brackets were considered and analysed. In orthodontic tooth movement, sliding resistance describes the interaction of the archwire with a bracket and depends on a material-specific coefficient of friction of the bracket material, the contact pressure by the ligature or the surface roughness of the bracket and archwire material. This sliding resistance opposes an orthodontically applied force and reduces it to various extents. This reduction was determined in the context of the investigations presented here.

According to Kusy and Whitley [23], sliding resistance consists of static or kinetic friction, due to the contact of the archwire with the bracket surface, binding, caused by bending of the archwire with resulting contact to the bracket corners and notching, when permanent deformations of the archwire at the bracket corners lead to stopping the tooth movement. It has to be mentioned that in the majority of clinical situations kinetic friction is irrelevant for orthodontic tooth movement and mainly binding and notching are of concern [7]. These binding and notching effects depend on a critical contact angle between the archwire and the bracket, which, in turn, depends additionally on their manufacturing precision. As a consequence, inaccuracies of the bracket slot size or archwire dimension influence the sliding resistance of the archwire–bracket complex. There are several studies dealing with this manufacturer-related precision [3, 24]. Dalstra et al. investigated the torque play with one and the same archwire type as a measure for slot precision in conventional and self-ligating brackets with the result that the actual torque play was larger than the theoretical one due to oversized slots for several bracket types [11]. Thus, an oversized slot influences the resistance properties of the archwire–bracket complex and it should be kept in mind that sliding behaviour depends not only on the bracket material properties. For this reason, the German Standard Institute has published a DIN standard for orthodontic archwires (DIN 3971) as well as for brackets and tubes (DIN 13971-2) in order to reduce these inaccuracies [12, 13]. These regulations describe the dimensions of orthodontic archwires and brackets within their tolerance limits. Joch et al. [18] also investigated the torque play in conventional and self-ligating brackets with the conclusion that for most brackets the actual torque play is larger due to oversized slots or the inability of self-ligating brackets to press the archwire into the bottom of the slot. Based on this, the knowledge of the bracket precision, more precisely the actual slot size, is a precondition for the comparative evaluation of sliding properties of different bracket types. This can be achieved by measuring the slot size for example using a microscope or by measuring the torque play values for all brackets with the same type of archwire.

Within the investigation here, the slot dimensions of each bracket tested were measured with the help of a digital light microscope. It has to be mentioned that the accuracy of these slot measurements depends on the individual definition of the vertical slot limitations done by the experimenter. In spite of this fact, as long as the measurements are performed under standardized conditions, the results can be compared to each other. In this investigation, all measurements of the slot dimensions were carried out under the same conditions and by the same experimenter. These measurements showed that the Brillant® polymer brackets as well as the Inspire Ice™ ceramic brackets had the highest manufacturer precision with 1–2% deviation from DIN standard. This background could explain the high friction values of the Inspire Ice™ ceramic brackets compared to all other brackets. Although the Brillant® polymer brackets showed a similar slot precision, the least resistance levels were found for this bracket type, which could be explained on the one hand by the low coefficient of friction of this bracket material and on the other hand by the low material resistance to mechanical strain, which is clearly visible due to the material abrasions at the mesial slot entry. The deformability of the slot limitations could also result in reduced binding and notching effects. As already shown in previous studies, the highest values of force loss due to resistance were also detected for ceramic brackets and here higher for the monocrystalline than for the polycrystalline ones [1, 5]. This could be related not only to the high precision of the slot fabrication, but also to the very sharp-edged design of the slot walls of the monocrystalline brackets because monocrystalline and polycrystalline ceramic brackets are subject to different manufacturing processes. In contrast to the polycrystalline brackets, the slot is milled into monocrystalline brackets and therefore has very sharp-edged limitations. Experimental studies could demonstrate the influence of the bracket bevel design on sliding resistance [9, 28]. They found that resistance could be reduced by increasing the bracket bevel angle. In addition, it was an important finding that the 3D-printed polymer Shark SL brackets showed partly better sliding properties than the metal brackets although the slots were more accurate than the metal brackets slots. These characteristics could possibly be attributed to the adequate material properties of this 3D-printed composite bracket type. The light and scanning electron microscope images showed a rather smooth surface and barely detectable defects after mechanical stress in the context of powder polishing.

If considering the possibility of combining alternative archwire materials with the brackets examined here, previous studies have shown that titanium–molybdenum alloy archwires resulted in the highest resistance values, especially in combination with ceramic brackets, followed by nickel–titanium alloy archwires and stainless steel archwires with the least values [22, 27, 29, 32]. For this reason, the analyses presented here were carried out with a stainless steel archwire only.

Regarding the influence of powder polishing on sliding behaviour of the different bracket material types, the polymer brackets were only very slightly influenced by exposure time. No adverse effects were found for the 3D-printed polymer Shark SL brackets even after 60 s of polishing time. The Brillant® polymer brackets also showed only marginal effects on sliding behaviour after 60 s polishing time. Although this bracket type had one of the highest slot precision, it showed the lowest resistance. Because of the fact that a kind of material shift could be detected at the mesial slot area in the microscopic images, the hypothesis can be supported that the advantageous sliding properties are rather due to material weakness. In addition, distinct perforations of the slot bottom were detected after 60 s of exposure. Interestingly, all these types of damage do not seem to have a notable influence on sliding behaviour. However, clinical use may be questioned due to the material weakness.

The ceramic brackets, and especially the monocrystalline Inspire Ice™ ones, showed the highest resistance values for the unpolished brackets in combination with the 0.016 inch × 0.022 inch archwire. Large chipping effects were detectable even in the unpolished brackets, indicating a pronounced clamping of the archwire with the highly precise slot limitations in the sense of some kind of notching.

The discovery® metal brackets showed the least mechanical effects from powder polishing of all brackets examined, although a trend towards reducing resistance was noticed. Similar to the 3D-printed polymer Shark SL brackets, these brackets showed the least visible effects in the light microscopic images. The surface of the scanning electron images showed a characteristic pattern which can be related to the sintering process, which is followed by recrystallisation as part of the manufacturing process. The electron microscopic images of the brackets after 60 s of polishing time showed a slight levelling of this pattern, indicating that this general powder polishing effect could reduce resistance after already 20 s of polishing time.

In the case of the titanium equilibrium® ti brackets, isolated chipping or abrasion were found on the slot bottom and entry, respectively. For this bracket type, a trend towards increased friction values after 60 s of polishing time could be observed. Perhaps, the surface roughness in combination with binding effects may have led to this increased sliding resistance.

In summary, it should be mentioned that the results discussed here are from an in vitro analysis. In clinical use, powder polishing is a rather dynamic process. In this study, powder polishing was additionally considered as a kind of material stress in order to compare the mechanical properties of different bracket materials. Furthermore, OMSS represents only a technical model for a simulated tooth movement, in which staining or dental calculus of the bracket–archwire complex, resistance due to root movement through the bone or masticatory forces are not taken into account. Future studies could also investigate the influence of the powder material composition or the particle size as well as the impact of speed or the powder–water ratio.

## Conclusions

- Although powder polishing with sodium bicarbonate can cause detectable defects on the bracket surface, the effect on sliding resistance is mostly insignificant or rather positive. Especially with regard to the improved sliding properties of a cleaned bracket–archwire complex, a regular and properly performed powder polishing should be performed.
- The Brillant® polymer brackets were characterized by low resistance values, but at the same time showed insufficient abrasion stability.
- Due to the fact that the Inspire Ice™ ceramic brackets showed both the highest resistance values and most chipping effects, it was the least qualified bracket type for sliding mechanics in this investigation.
- Modern 3D-printed polymer brackets provided better sliding properties than commonly used metal brackets, even with a higher slot precision.
